# An Ontology Proposal for Implementing Digital Twins in Hospitality: The Case of Front-End Services

**DOI:** 10.3390/s25144504

**Published:** 2025-07-20

**Authors:** Moises Segura-Cedres, Desiree Manzano-Farray, Carmen Lidia Aguiar-Castillo, Rafael Perez-Jimenez, Victor Guerra-Yanez

**Affiliations:** 1 Instituto para el Desarrollo Tecnológico y la Innovación en Comunicaciones (IDeTIC), Universidad de Las Palmas de Gran Canaria (ULPGC), Juan de Quedasa 30, 35001 Las Palmas de Gran Canaria, Spain; moises.segura101@alu.ulpgc.es (M.S.-C.); desiree.manzano@ulpgc.es (D.M.-F.); lidia.aguiar@ulpgc.es (C.L.A.-C.); 2 Pi Lighting, 1950 Sion, Switzerland; victor.guerra@pi-lighting.com

**Keywords:** digital twins, ontology, hotel receptionist, human-centric AI, front office operations, hospitality industry

## Abstract

The implementation of Digital Twins (DTs) in hospitality facilities represents a significant opportunity to optimize front-end services, enhancing guest experience and operational efficiency. This paper proposes an ontology-driven approach for DTs in hotel reception areas, focusing on integrating IoT devices, real-time data processing, and service optimization. By modeling interactions between guests, receptionists, and hotel management systems, DTs enhance resource allocation, predictive maintenance, and customer satisfaction. Simulations and historical data analysis enable forecasting demand fluctuations and optimizing check-in/check-out processes. This research provides a structured framework for DT applications in hospitality, validated through scenario-based simulations, showing significant improvements in check-in time and guest satisfaction. Validation was conducted through scenario-based simulations reflecting real-world operational challenges, such as guest surges, room assignment, and staff workload balancing. Metrics including check-in time, guest satisfaction index, task completion rates, and prediction accuracy were used to evaluate performance. Simulations were grounded in historical hotel data and modeled typical peak-period dynamics to ensure realism. Results demonstrated a 25–35% reduction in check-in time, a 20% improvement in staff efficiency, and significant enhancements in guest satisfaction, underscoring the practical value of the proposed framework in real hospitality settings.

## 1. Introduction

The hospitality industry is undergoing a profound digital transformation, driven by the adoption of advanced technologies to improve both operational efficiency and guest satisfaction [1,2,3]. Among these technologies, Digital Twins (DTs), virtual replicas of physical environments that enable real-time monitoring, predictive analytics, and intelligent decision making, have emerged as a promising but underutilized tool in front-end hotel services. Front-end hotel services refer to guest-facing operations such as check-in/check-out, concierge support, room assignment, and lobby interactions—functions that directly shape the customer experience. Despite their potential, DTs have been underutilized in these contexts due to high implementation costs, integration complexity with legacy systems, and limited awareness of their applicability beyond industrial domains [4,5].

Originally developed for industrial and smart city applications [6,7,8,9,10], DTs are now gaining relevance in service-oriented domains [11,12]. In hotel reception areas, they enable dynamic resource allocation, improved check-in/check-out processes, and proactive guest service. These capabilities are driven by the integration of data from IoT devices, booking systems, and guest interaction platforms [13,14,15,16,17].

**Figure 1:**
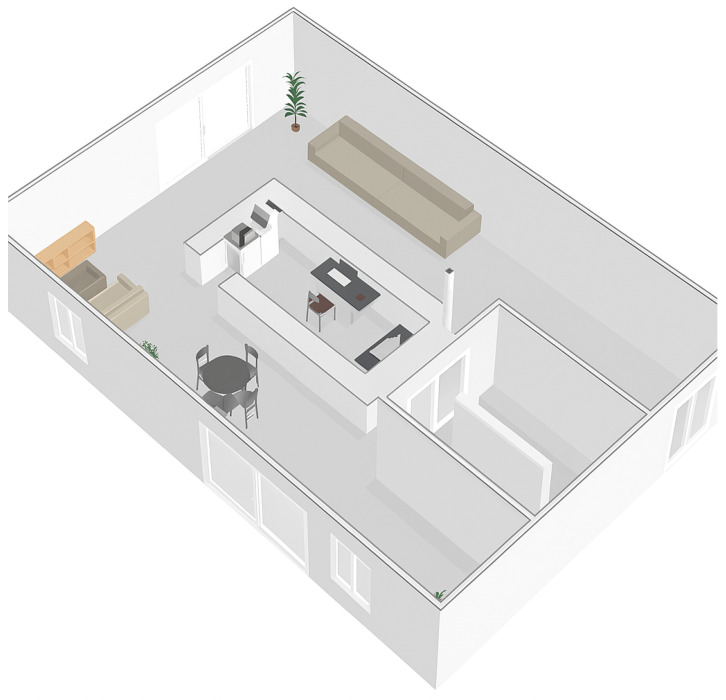
Sample reception image.

In Figure 1 you can see the recreation of a hotel reception.

However, the implementation of DTs in hospitality requires more than technological replication. Unlike industrial settings that focus on mechanical systems, hospitality environments are inherently human-centered, involving social interactions, emotional dynamics, and personalized service expectations. As such, effective DTs must incorporate psychological and behavioral modeling to capture guest preferences, staff workload patterns, and cognitive-emotional responses. Insights from cognitive psychology and behavioral economics can enhance these models, allowing DTs to support proactive service adaptation and stress-aware task distribution among front-desk employees [10,18,19,20,21].

Equally important are the ethical implications of deploying AI-powered DTs in guest-facing scenarios. To address these challenges, issues such as data privacy, algorithmic bias, and transparency must be proactively managed to ensure regulatory compliance and build user trust. Techniques such as explainable AI (XAI), digital identity management, and data anonymization can help mitigate risks associated with surveillance, consent, and fairness [22,23,24].

**Figure 2:**
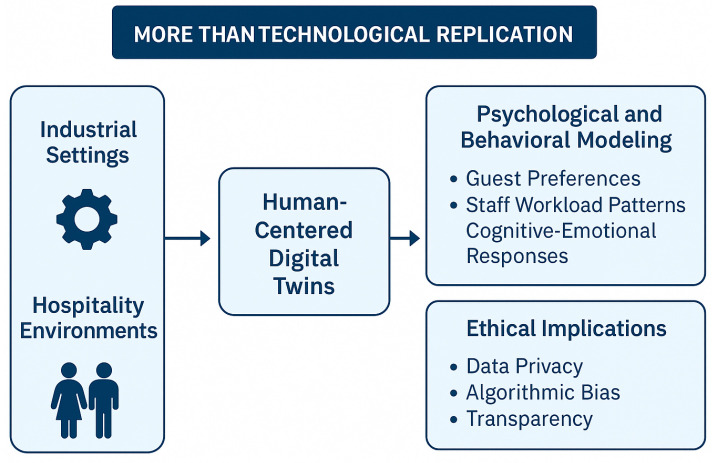
Human-centered implementation of DTs in hospitality.

Figure 2 visually articulates the conceptual transition from traditional DT applications in industrial settings to their emerging role in human-centered hospitality environments. Unlike industrial DTs that focus primarily on monitoring mechanical assets and optimizing technical operations, this type of DT prioritizes psychological, behavioral, and ethical dimensions. The diagram emphasizes that hospitality environments require systems capable of modeling guest preferences, understanding staff workload patterns, and interpreting cognitive-emotional states. These features enable personalized and empathetic service delivery, distinguishing them from purely efficiency-driven industrial models. Furthermore, the illustration highlights the ethical implications inherent in applying AI and DTs in human-facing contexts. Issues such as data privacy, algorithmic bias, and transparency are not peripheral concerns but central design requirements. By framing DTs as socio-technical systems, the diagram reinforces the idea that effective deployment in hospitality requires more than technological replication—it demands a holistic integration of human, ethical, and contextual factors.

Artificial intelligence (AI) further amplifies the potential of DTs in hospitality by enhancing automation, decision-making, and predictive capabilities. AI-powered DTs can process vast amounts of structured and unstructured data, including reservation records, IoT telemetry, and social media sentiment, to optimize front-desk operations [1,3].

Key applications include predictive analytics to forecast guest arrivals and demand fluctuations, intelligent virtual assistants for booking support, and computer vision systems that streamline check-in procedures through facial recognition and behavior monitoring [25]. AI also enables anomaly detection, identifying irregular patterns in guest behavior or operational workflows. For instance, if a guest consistently requests services at unusual hours, the system might proactively recommend targeted promotions or raise security alerts.

The synergy between AI and DTs creates a robust cognitive layer that supports real-time, adaptive service delivery. Facial recognition and sentiment analysis enhance personalization, while virtual assistants powered by natural language processing facilitate guest interaction and automate routine inquiries. The bidirectional linkage between AI and DTs allows AI to enrich the DT’s situational awareness, while the DT provides structured, real-world context for intelligent reasoning. This integration results in a responsive, personalized, and efficient digital representation of front-desk operations [22].

**Figure 3:**
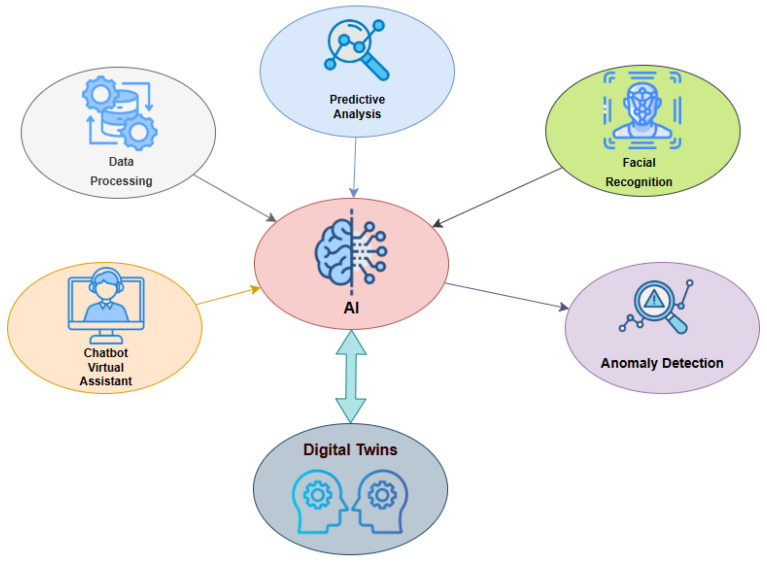
Artificial intelligence in DTs.

Figure 3 illustrates the integration of artificial intelligence (AI) and DT technologies within a service-oriented framework, highlighting the AI-driven functionalities that enhance DT performance. AI acts as a central engine enabling multiple intelligent capabilities, including data processing, predictive analysis, facial recognition, anomaly detection, and chatbot-based virtual assistants. These components enrich the situational awareness and decision-making capacity of DTs by providing real-time insights and adaptive responses. For instance, predictive analysis supports operational forecasting, while anomaly detection identifies irregular behaviors in guest flow or facility conditions. Facial recognition and sentiment analysis can personalize services and trigger proactive interventions. Simultaneously, virtual assistants powered by natural language processing facilitate guest interaction and automate routine inquiries. The bidirectional linkage between AI and DTs demonstrates a synergistic relationship in which AI enhances the cognitive layer of the system, while the DT provides structured, real-world context for intelligent reasoning. This configuration enables a responsive, personalized, and efficient digital representation of front-desk operations.

To address these multifaceted challenges, this paper proposes an ontology-based DT framework tailored to hotel reception services. Ontology, in the context of computer science and artificial intelligence, refers to a formal and explicit specification of a shared conceptualization of a domain. It defines a set of entities, attributes, relationships, and constraints that describe the knowledge structure of a particular system or environment [26,27,28,29].

The framework leverages semantic modeling to ensure interoperability between heterogeneous systems and supports real-time reasoning for adaptive service delivery. It integrates formal ontologies with a modular DT platform capable of ingesting real-time data, supporting semantic inference, and enabling intelligent automation [26,30]. Artificial intelligence components enhance the system’s ability to perform predictive analytics, facilitate sentiment-sensitive interactions, and enable context-aware decision-making.

By aligning technical innovation with human-aware design and ethical governance, this research advances a new paradigm for smart hospitality—one where DTs are not only operational tools but also socio-technical systems that enhance service quality, employee engagement, and guest satisfaction.

## 2. Background and Related Work

### 2.1. DTs in Hospitality

DTs have traditionally been associated with industrial and engineering domains, where they serve as virtual representations of physical systems to enable real-time monitoring, simulation, and predictive analytics [6,7,8,9,10]. However, the core principles of DTs—data integration, dynamic modeling, and intelligent decision-making—are increasingly being translated into service-oriented sectors, particularly the hospitality industry [13,14,15,16,17] .

In hospitality, DTs are reshaping how hotels manage both operational processes and customer experiences. By creating a virtual counterpart of the physical hotel environment, including its infrastructure, guest-facing services, and backend operations, DTs provide a holistic view of system performance. These models are continuously updated through data streams from IoT devices, guest interactions, building management systems, and reservation platforms [31].

One of the key applications of DTs in hospitality is in front-desk operations, where they facilitate automated check-in/check-out, predictive resource allocation, and real-time guest flow management. For instance, a DT can monitor the number of guests approaching the reception area and dynamically adjust staffing levels or activate self-service kiosks to prevent bottlenecks. Furthermore, DTs can integrate historical booking data and external factors—such as flight delays or weather conditions—to forecast guest arrivals and prepare personalized services in advance [10].

Another critical function lies in room and facility management. DTs can track room conditions—such as temperature, occupancy, and maintenance needs—by processing sensor data in real time. This enables proactive issue resolution, such as scheduling HVAC repairs before guests are affected, or optimizing energy usage by adjusting systems based on occupancy patterns [32].

Additionally, DTs enhance guest experience personalization. Using data from previous stays, preferences, loyalty programs, and, where ethically and legally appropriate, social media interactions, DTs can tailor services to individual guests. For example, a returning guest may find that their preferred room temperature, lighting, and welcome message are already set upon arrival, all orchestrated through the DT model. This level of personalization fosters stronger guest loyalty and differentiates service quality in a competitive market [33].

Emerging research also explores the role of DTs in event and conference management within hotel facilities. In this context, DTs can support the planning and coordination of large-scale events by simulating room configurations, optimizing equipment allocation, and scheduling staff based on anticipated attendee flow. They can also model crowd movement to identify potential congestion points and recommend spatial arrangements that enhance both operational efficiency and guest safety [34].

Despite their growing potential, the implementation of DTs in hospitality faces several challenges. These include integration with legacy Property Management Systems (PMSs), ensuring data privacy and guest consent, and maintaining scalability across multiple hotel branches. Moreover, many current DT implementations remain data-centric, lacking the semantic structure required for cross-system interoperability and reasoning [22,35].

To address these issues, recent approaches advocate for the incorporation of ontology-based modeling and human-centric AI design principles [36,37], allowing DTs to function not only as operational tools but also as adaptive, context-aware systems that understand and respond to human behavior. The convergence of AI, IoT, and semantic web technologies marks a significant transformation in how hospitality environments are managed and experienced.

### 2.2. Human-Centric AI

Human-centric artificial intelligence (HCAI) is an emerging paradigm that emphasizes the development of AI systems aligned with human values, cognitive abilities, emotional awareness, and social context. Unlike traditional AI models that prioritize efficiency and optimization in isolation, HCAI approaches seek to increase human decision-making, foster collaboration, and promote transparency and ethical design principles in technological applications [18,19,20,21].

This paradigm is particularly relevant in the hospitality sector, where service quality and interpersonal interaction are central to the guest experience. Unlike industrial DTs that primarily manage mechanical systems or logistics, hospitality DTs must interpret subtle emotional cues, behavioral patterns, and social interactions in real time. Guests expect empathetic, personalized, and context-sensitive services that adapt fluidly to their needs—demand capabilities such as real-time emotional interpretation, adaptive service delivery, and ethical responsiveness—functions that traditional AI systems typically lack without this type of design [38].

In practical terms, a user-adaptive digital replica in a hotel reception can incorporate emotion recognition from facial expressions, sentiment analysis of guest feedback, or voice tone classification to evaluate guest satisfaction or frustration. These signals can dynamically trigger personalized service responses, such as alerting staff when human intervention is required or offering customized rewards based on detected positive sentiment. However, because emotional and behavioral data are highly sensitive, their collection and analysis raise significant privacy concerns. Ensuring explicit user consent, secure data handling, and transparency in how such insights are used is critical to maintaining trust and ethical compliance [39,40,41,42].

Beyond personalization, HCAI also addresses pressing ethical concerns in AI deployment, such as bias, accountability, and data privacy. It promotes the use of explainable AI (XAI) models that provide transparent, interpretable reasoning paths, making it easier for both staff and guests to understand how decisions are made [43]. Additionally, HCAI emphasizes fairness and inclusivity through bias mitigation techniques [44,45], and the protection of user data through privacy-preserving mechanisms [46,47]. These measures contribute to greater trust, informed consent, and regulatory compliance in AI-supported service environments [48,49,50].

Importantly, HCAI enhances not just the guest experience, but also the working environment for hotel staff. By transforming DTs into collaborative systems—rather than automated task enforcers—HCAI empowers employees through decision support dashboards that offer real-time insights on service prioritization, task allocation, and issue resolution. This not only improves response effectiveness but also reduces cognitive overload and fosters job satisfaction by allowing staff to focus on meaningful, human-aware service interactions [51,52,53].

In summary, integrating HCAI into DT systems ensures that the deployment of advanced technologies in hospitality respects and reinforces the core values of empathy, transparency, trust, and personalization. Far from replacing the human touch, this approach enhances both guest engagement and staff empowerment, setting a new standard for ethical and adaptive AI in service-oriented environments [54].

**Figure 4:**
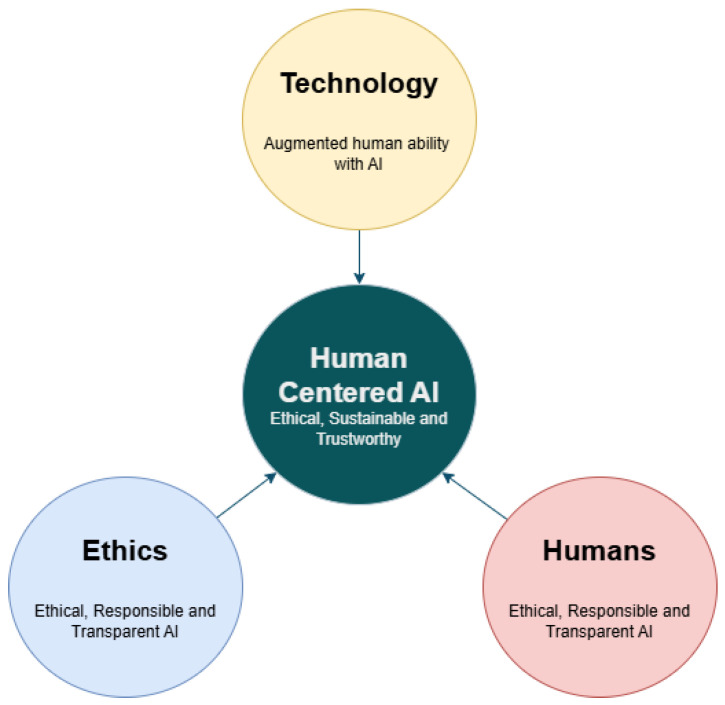
Human-centered AI involves different aspects such as technology, ethics, and human factors (based on [18]).

Figure 4 illustrates the conceptual shift from traditional DT implementations in industrial settings to human-centered DTs tailored for hospitality environments. While industrial DTs primarily focus on replicating and optimizing mechanical processes, hospitality applications demand a broader scope that integrates psychological, behavioral, and ethical dimensions. This approach emphasizes modeling guest preferences, staff workload patterns, and cognitive-emotional responses to deliver adaptive and empathetic services. Moreover, it accounts for critical ethical considerations such as data privacy, algorithmic bias, and system transparency. This expanded framework moves beyond mere technological replication, positioning DTs as socio-technical systems that respond to human needs and values in dynamic service environments.

### 2.3. Ontology in Smart Environments

Ontology, in the context of computer science and artificial intelligence, refers to a formal and explicit specification of a shared conceptualization of a domain [26]. It defines a set of entities, attributes, relationships, and constraints that describe the knowledge structure of a particular system or environment. In smart environments—such as intelligent buildings, connected homes, and digitally enabled hotels—ontologies provide a semantic backbone that enables interoperability, reasoning, and dynamic knowledge integration [37,55].

Smart environments typically consist of heterogeneous systems, including IoT sensors, building management platforms, user interfaces, and AI-driven analytics. Without a common vocabulary and structure, these systems struggle to share information effectively. Ontologies address this by offering a shared schema that aligns sensor outputs, user data, and system logic into a coherent, machine-interpretable framework [56,57].

In the hospitality industry, ontology-based modeling is especially useful due to the diverse and dynamic nature of service operations. Hotel front-desk services, for instance, involve managing reservations, room status, guest interactions, staff schedules, and environmental controls. An ontology for this domain might define entities such as Guest, Room, Receptionist, and Device, along with relationships like checksInTo, monitors, or requestsService. This enables DTs to contextualize real-time data, detect semantic patterns, and infer optimal actions [58].

Beyond static representation, ontologies also support inference and reasoning. For example, if a guest is detected to have a mobility impairment (based on past preferences or feedback), the ontology can infer the need for a ground-floor room, proximity to elevators, or staff assistance. These insights are not pre-programmed as fixed rules; instead, they are inferred dynamically through logical reasoning applied to the ontology’s semantic structure [59,60].

Moreover, ontologies facilitate data interoperability and scalability. By adopting standard formats like OWL (Web Ontology Language), hotel systems can integrate with external platforms, such as booking engines, CRM systems, or city-level smart infrastructure—without the need for rigid data transformation [61,62].

Although domain-specific ontologies such as Hontology (for tourism) and RealEstateCore (for smart buildings) offer valuable templates that can be adapted or extended for the hospitality context [57,63], they lack the granularity and operational semantics required for real-time front-desk service delivery. Specifically, these models often do not represent dynamic staff-guest interactions, workload balancing, or feedback-driven service adaptation—elements that are essential for human-centric DTs in hospitality. Furthermore, many existing ontologies are static and do not support reasoning over live IoT telemetry or predictive analytics, limiting their suitability for responsive and intelligent service environments. Our ontology addresses these gaps by integrating behavior modeling, service task orchestration, and semantic interoperability mechanisms tailored for hospitality workflows.

By grounding DT implementations in ontology-based frameworks, hotels can ensure not only accurate data representation but also meaningful automation, adaptive behavior (for instance, if a guest frequently requests additional towels during previous stays, the system can automatically schedule a delivery upon their check-in, without requiring a new request—demonstrating proactive service enabled by behavioral inference.), and intelligent service delivery—core pillars of next-generation smart hospitality environments [57].

A notable real-world application of ontology in smart environments is the Autonomic Semantic-Based Context-Aware Platform for Mobile Applications in Pervasive Environments [64]. This platform leverages ontologies to interpret context data dynamically in pervasive computing scenarios, enabling mobile systems to adapt their behavior based on user location, device status, and environmental changes. The system demonstrates how semantic reasoning can facilitate intelligent context-awareness in real-time applications, illustrating the versatility and effectiveness of ontology-driven architectures beyond industrial use cases.

## 3. Problem Statement and Objectives

The hospitality sector is undergoing rapid digital transformation, yet the application of intelligent and interoperable systems, such as DTs, remains fragmented and underdeveloped, particularly in front-end hotel services. The hotel reception desk, which plays a pivotal role in shaping the guest experience, is often constrained by outdated technologies, siloed systems, and reactive service delivery models [65].

Traditional hotel front-desk operations typically rely on multiple independent systems for reservations, room management, customer interaction, and staff scheduling. These systems rarely communicate in real-time, limiting the ability of hotel personnel to respond dynamically to fluctuating guest demands, occupancy rates, or environmental conditions. As a result, issues such as long check-in queues, inefficient room allocation, and misaligned staffing schedules persist, negatively impacting guest satisfaction and operational efficiency [66].

Furthermore, while data is increasingly available through IoT devices, building management systems, and digital guest platforms, most hotels lack a semantic framework—a formal structure that links disparate data sources using machine-interpretable meanings. For example, without a semantic model, a temperature reading from a sensor, a guest preference stored in a CRM, and a maintenance request are treated as isolated data points rather than connected knowledge. A semantic framework allows the system to infer that a guest in Room 210 who prefers cooler environments and submits a comfort complaint during check-in may require HVAC adjustment to integrate and interpret this data contextually.

The absence of such a framework hinders the deployment of intelligent applications such as real-time anomaly detection (e.g., identifying malfunctioning elevators from sensor and service request data), personalized service planning (e.g., adjusting room assignments based on past preferences), and predictive workload forecasting (e.g., reallocating reception staff based on guest arrival trends). Without semantic interoperability, AI models cannot perform reliable reasoning, pattern recognition, or contextual adaptation—core capabilities required for a responsive and efficient front-desk operation [66].

Incorporating DTs into front-desk operations presents a significant opportunity to overcome these limitations. However, both the academic literature and commercial solutions exhibit a gap in domain-specific methodologies tailored to front-desk hospitality operations—this includes a lack of technical frameworks that account for real-time semantic interoperability and human-aware service delivery. Specifically, there is a gap in using ontology-based modeling to support semantic interoperability and real-time reasoning across heterogeneous systems. Additionally, the integration of HCAI—designed to support both guests and staff—is often overlooked in technical implementations of DTs.

Therefore, this research addresses the following core problem:

*How can an ontology-driven DT framework be designed and implemented to optimize hotel front-desk operations, enabling real-time data integration, predictive service delivery, and human-centered interaction?*

To answer this question, the study sets forth the following specific objectives:

- Develop a domain-specific ontology for hotel front-desk operations using formal semantic modeling in OWL (Web Ontology Language). OWL was selected due to its expressiveness, reasoning capabilities, and compatibility with existing semantic web standards, which are essential for enabling interoperability and inference over heterogeneous hotel data.
- Integrate real-time data sources—including sensor telemetry, reservation systems, and guest interaction logs—into a unified DT environment.
- Implement AI-powered services within the DT framework. These include predictive analytics for guest flow forecasting, dynamic task allocation based on receptionist workload levels, sentiment-driven service recommendations, and anomaly detection in operational workflows.
- Support human-centric interaction design by incorporating feedback loops, facial expression, and voice-based sentiment analysis, and staff assistance tools to enhance both guest experience and employee engagement.
- Validate the proposed framework through scenario-based simulations and quantitative performance metrics. Scenarios will include dynamic check-in surges, room reassignment, and staff reallocation events. Evaluation metrics will encompass average check-in time, guest satisfaction index, staff task efficiency, and reasoning accuracy under real-time conditions.

This study aims not only to propose a technically robust DT architecture but also to demonstrate its value as a socio-technical system—capable of improving both operational outcomes and human interactions in the hospitality domain.

**Figure 5:**
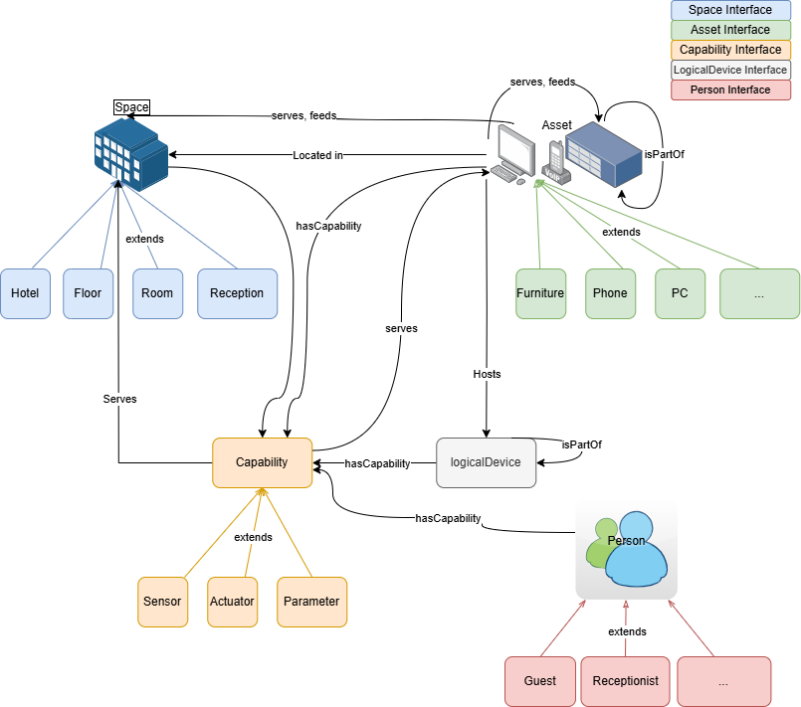
Domain-specific ontology for hotel front-desk operations.

Figure 5 presents a semantic model for DT environments in hospitality, based on a modular ontology structure. The model is organized around five core interfaces: Space, Asset, Capability, LogicalDevice, and Person. The Space interface represents the physical layout of the environment and includes hierarchical elements such as Hotel, Floor, Room, and Reception, with semantic relationships such as serves, feeds, and located in used to link physical spaces and operational functions. The Asset interface encompasses tangible objects such as furniture, phones, and PCs, which are part of a broader Asset class and extend specific physical roles. These assets are associated with Capabilities—including sensors, actuators, and parameters—that define functional attributes and service roles. LogicalDevice elements are abstracted system components that host or consume capabilities and bridge physical devices with semantic logic. The Person interface captures human actors in the system, such as Guests and Receptionists, and links them to the environment via service interactions and assigned capabilities. Overall, the model enables semantic interoperability, behavioral modeling, and system-level reasoning by clearly representing the relationships between spaces, devices, people, and functionalities in a hotel DT context.

## 4. Ontology-Based DT Framework

The development of the proposed ontology was grounded in established semantic modeling practices and was specifically inspired by the structure and principles of the Open DTs Smart Cities Ontology developed by Microsoft Azure [67]. This publicly available ontology provides a modular and extensible framework for modeling urban environments and infrastructure using DTDL. Its emphasis on interoperability, standardized property sets, and clear relationship modeling served as a foundational reference for adapting domain-specific classes and properties relevant to hotel reception environments. This approach allows for the seamless integration of heterogeneous data sources—such as IoT sensor data, guest profiles, and reservation records—into a cohesive, machine-readable model that supports real-time monitoring and decision-making.

### 4.1. Conceptual Overview

**Figure 6:**
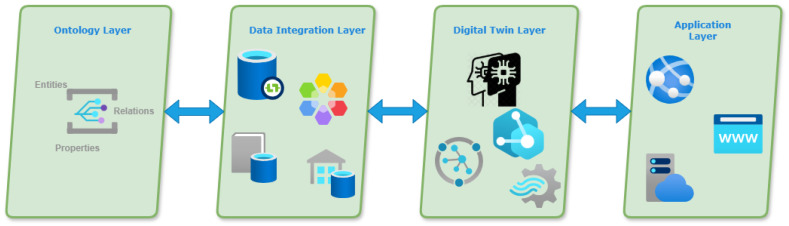
Ontology framework.

As we can see in Figure 6, the framework is structured into four interconnected layers:

- Ontology Layer: Defines the semantic model of the domain, including entities (e.g., Guest, Room, Reservation), relationships (e.g., checksInTo, monitors, assignedTo), and properties (e.g., room status, guest preferences).
- Data Integration Layer: Aggregates data streams from IoT sensors, Property Management Systems (PMSs), Customer Relationship Management (CRM), and booking engines. These data are annotated and aligned with the ontology using semantic mapping techniques.
- DT Layer: This layer instantiates the semantic model dynamically and maintains real-time representations of physical and logical entities. It is responsible for handling state updates, managing the twin graph structure, and enabling interactions through standardized APIs. The implementation is compatible with any DT platform that supports semantic modeling, real-time data integration, and event-driven architecture.
- Application Layer: Comprises service modules such as predictive analytics, anomaly detection, and staff decision support tools. These applications query the twin graph and ontology to deliver insights and automation.

Each layer is interdependent and communicates via standardized data protocols and interfaces to ensure seamless interaction and synchronization across components. The framework utilizes MQTT (Message Queuing Telemetry Transport) for real-time telemetry transmission between IoT devices and middleware services, enabling lightweight, publish–subscribe messaging. RESTful APIs (Representational State Transfer Application Programming Interface) are employed for integration with external systems such as PMS and CRM platforms, ensuring accessible and platform-agnostic data exchange.

Semantic queries and knowledge extraction are performed using SPARQL (SPARQL Protocol and RDF Query Language), allowing dynamic reasoning over RDF (Resource Description Framework) data stores. Additionally, the ontology layer adheres to semantic web standards including OWL (Web Ontology Language) and RDF, ensuring formal and interoperable knowledge representation. Together, these protocols establish a modular and scalable infrastructure capable of supporting real-time decision-making in a complex hotel environment.

**Figure 7:**
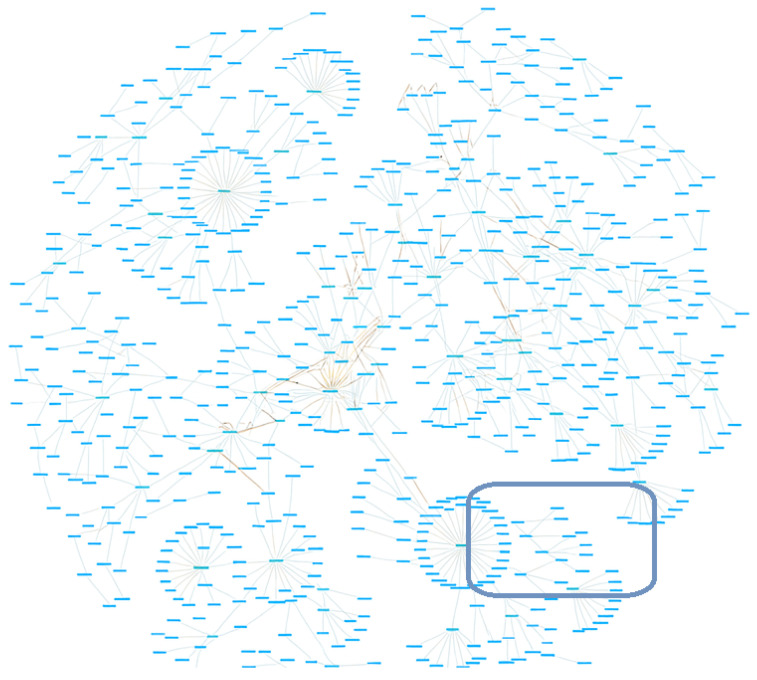
Full project Ontology.

Figure 7 presents the complete ontology graph developed for the DT framework applied to front-desk operations in hospitality environments. Each node in the graph represents a semantic entity—either a class, individual instance, or property—defined using OWL 2.0 and interconnected through logical relationships and object properties. The structure demonstrates the modularity and expressiveness of the ontology, with visible clusters corresponding to key domains such as Guest, Room, Reservation, Receptionist, and IoTDevice. Radial subgraphs illustrate class hierarchies, while denser regions reflect high-connectivity areas involving reasoning and contextual inference (e.g., service rules, sensor-driven alerts, and task assignments). The centralized connectivity underscores the ontology’s role as a unifying semantic layer, enabling interoperability across systems and real-time AI-driven decision support. This comprehensive semantic structure supports dynamic instantiation, adaptive service behavior, and human-centric logic within the Digital Twin ecosystem.

**Figure 8:**
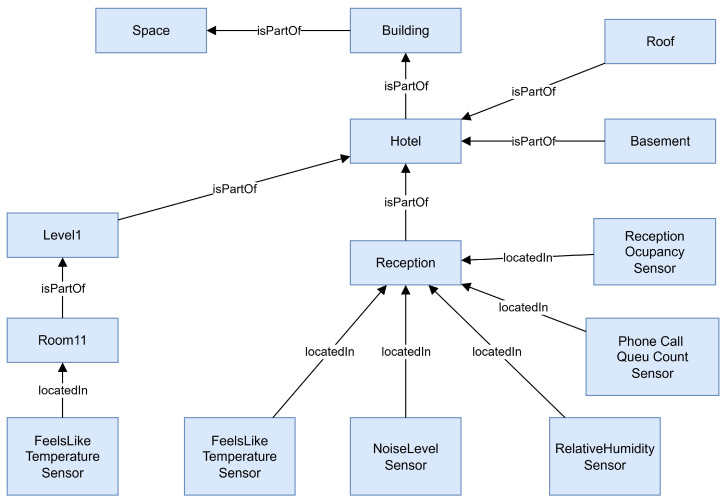
Detail from Figure 7, about the recepcionist interactions.

Figure 8 shows a detailed segment of the overall ontology focused on the class Receptionist, illustrating its position within the semantic network of front-desk operations. The Receptionist is semantically classified as a subclass of Person, inheriting general human-related attributes while introducing specialized roles and relationships. Yellow arrows denote object properties, such as includesRole, hasMember, and isMemberOf, linking individuals to organizational units like Department or Company. The green lines indicate class hierarchies and inheritance, situating Receptionist in the broader ontological structure that includes entities like Guest, Sensor, Event, and LeaseContract. This interconnected view emphasizes the receptionist’s role as both a service actor and a contextual entity, participating in workflows triggered by sensor data, event occurrences, and organizational policies—reinforcing the ontology’s capacity to model real-time, human-centric interactions in hotel environments.

#### Formal Reasoning Rules (SPARQL)

To support semantic inference in real-time hotel operations, we defined rules using SPARQL queries and filters. These rules allow the system to extract contextual knowledge from RDF graphs and trigger decisions based on logical conditions. Below are selected examples:

# Rule 1: Identify guests who are checking in today

PREFIX : <http://example.org/hotel#>

PREFIX xsd: <http://www.w3.org/2001/XMLSchema#>

**

SELECT ?guest WHERE {

?guest a :Guest ;

:makesReservation ?res .

?res :checkInDate "2025-06-15"^^xsd:date .

}

**

# Rule 2: Trigger housekeeping if a room is assigned and needs cleaning

SELECT ?room WHERE {

?room a :Room ;

:hasStatus "needsCleaning" ;

:isAssignedTo ?res .

}

**

# Rule 3: Recommend task redistribution for overloaded staff

SELECT ?receptionist WHERE {

?receptionist a :Receptionist ;

:workloadLevel "high" .

}

### 4.2. Ontology Design and Components

The ontology was developed using the Web Ontology Language (OWL), following the best practices described by Noy and McGuinness [55]. It models the semantic structure of hotel reception operations through the explicit definition of classes, properties, attributes, and relationships. Classes represent the core concepts or entities within the domain—such as Guest, Room, Reservation, Receptionist, and IoT Device—each encapsulating a unique set of roles and behaviors in the reception environment. Properties describe the relationships or connections between classes (object properties), as well as data values associated with individual instances (data properties). For instance, the Guest class includes the object property makesReservation, linking it to the Reservation class, and data properties like hasPreference or hasFeedback to capture user-specific attributes. Attributes such as checkInDate and duration associated with Reservation, or deviceType and sensorData associated with IoT Device, serve to store domain-relevant data points. Relationships between these classes form a directed graph, enabling logical reasoning and service orchestration. For example, the ontology defines that if a Room instance has the status unoccupied and a Reservation is linked to a Guest with an imminent check-in, a preparationTask should be generated for the corresponding Receptionist. This modeling approach ensures semantic clarity, machine-interpretable knowledge representation, and operational adaptability, forming a solid foundation for intelligent, context-aware DT behavior in hospitality environments.

At the core of the ontology are five primary classes:

- Guest: This class represents the hotel customer and includes properties such as hasPreference, which stores personal preferences (e.g., preferred room type or amenities), hasFeedback, which links to guest satisfaction data or complaints, and makesReservation, which connects each guest to one or more reservation instances.
- Room: This entity reflects the physical hotel room and is defined by properties like hasStatus (e.g., occupied, ready, needs cleaning), isMonitoredBy (linking to IoT sensors), and isAssignedTo, which associates the room with a specific guest reservation.
- Reservation: The Reservation class serves as a temporal connector between guests and rooms. Key attributes include checkInDate and duration, which define the booking window and are essential for operational planning and room availability.
- Receptionist: This human actor is associated with service responsibilities. The class includes properties such as assignedTasks (e.g., check-in procedures, complaint resolution), handlesRequest (linking to guest service interactions), and workloadLevel, which allows the system to monitor and balance staff performance.
- IoT Device: Representing the various sensors and smart devices deployed throughout the facility, this class includes attributes like deviceType (e.g., temperature, occupancy), monitors (which links to rooms or environmental parameters), and sensorData, which captures live telemetry input.

#### Cardinalities and Class Relationships

To improve semantic precision, cardinalities were defined between key ontology classes, such as the following:

- A Guest can be linked to one or more Reservation instances via the makesReservation property (cardinality: 1..n), while each Reservation must be associated with exactly one Guest (1..1).
- Each Reservation is assigned to exactly one Room using the isAssignedTo property (cardinality: 1..1). A Room, however, can be associated with zero or more Reservation instances over time (0..n), allowing for reuse.
- A Receptionist can handle multiple GuestRequest instances via the handlesRequest property (cardinality: 0..n), and each GuestRequest is linked to one and only one Receptionist (1..1).
- Each IoTDevice monitors at least one Room through the monitors property (cardinality: 1..n), while a Room can be monitored by multiple IoTDevice instances (0..n), enabling layered sensing.

### 4.3. Semantic Integration with a DT Platform

The semantic integration is achieved through a modular DT platform that supports ontology-driven modeling. The OWL ontology is translated into a format compatible with the platform’s DT schema, ensuring that class structures, relationships, and properties are preserved and interpretable by the runtime system.

Each physical or logical entity in the reception environment, such as a guest room, a self-check-in kiosk, or a receptionist, is instantiated as a DT. Real-time telemetry from IoT devices updates the associated properties of these twins, enabling continuous synchronization with the physical environment. Relationships between entities are expressed through the platform’s twin graph or data model, capturing semantic links such as a guest being associated with their reservation and assigned room, or a sensor device monitoring specific environmental conditions. This approach ensures semantic interoperability, supports real-time reasoning, and enables dynamic service orchestration.

### 4.4. Predictive and Adaptive Capabilities

By combining the semantic expressiveness of the ontology with real-time data streams, the DT framework enables a range of advanced, intelligent functionalities. Through predictive analytics, the system can forecast guest arrival patterns, anticipate room turnover rates, and estimate service load by leveraging both historical records and live telemetry data. These insights support proactive resource planning and operational readiness. In parallel, adaptive interaction mechanisms allow the system to dynamically recommend task assignments to receptionists based on their current workload and availability, thereby improving task distribution and reducing response times. Additionally, the ontology supports contextual reasoning by interpreting operational situations in real time. For instance, it can infer that a guest checking in later than expected may require expedited service, or recognize that frequent service requests from a single room may indicate a broader systemic issue. These capabilities collectively enhance the system’s ability to deliver responsive, personalized, and efficient front-desk services.

### 4.5. Benefits and Innovation

This framework introduces several key innovations to the application of DTs in hospitality contexts. First, it establishes a formal semantic model specifically tailored to hotel reception environments, enabling structured representation of entities, interactions, and operational processes. By bridging the gap between physical infrastructure—such as sensors and IoT devices—and logical service layers through ontology-driven modeling, the system creates a cohesive, machine-interpretable environment. Additionally, it supports proactive and human-centric service delivery by integrating artificial intelligence and semantic web technologies, allowing for adaptive, real-time responses to both guest needs and staff workloads. The framework is also designed with scalability and reusability in mind, making it adaptable to various hotel types and chain-wide deployments. Collectively, these contributions position the ontology-based DT approach as a robust and intelligent foundation for shifting front-desk operations from reactive, manual processes to predictive, data-informed, and personalized service systems.

## 5. Implementation and Evaluation

The proposed ontology-based DT framework was implemented using a combination of semantic modeling tools, cloud-based platforms, and simulation environments. Ontology modeling was performed using Protégé, leveraging the OWL 2.0 specification for defining domain concepts, properties, and axioms.

For real-time data integration, the system utilized Node-RED and MQTT brokers to simulate and route telemetry from IoT sensors, guest interactions, and environmental conditions. These tools enabled event-driven orchestration and seamless communication between simulated devices and the DT graph.

The runtime DT layer was instantiated on a graph-based platform compatible with Azure DTs, supporting semantic modeling and API-driven state management. Real-time analytics and prediction services were deployed using Apache Kafka for data streaming and Spark Streaming for processing.

To simulate hotel scenarios and visualize system behavior, the team used Unity to render a 3D virtual hotel environment. Additionally, live system metrics and events were monitored through Grafana dashboards connected to backend services.

This modular architecture ensured that semantic logic, real-time data, and AI-driven services were tightly integrated and scalable across simulated hotel environments.

**Figure 9:**
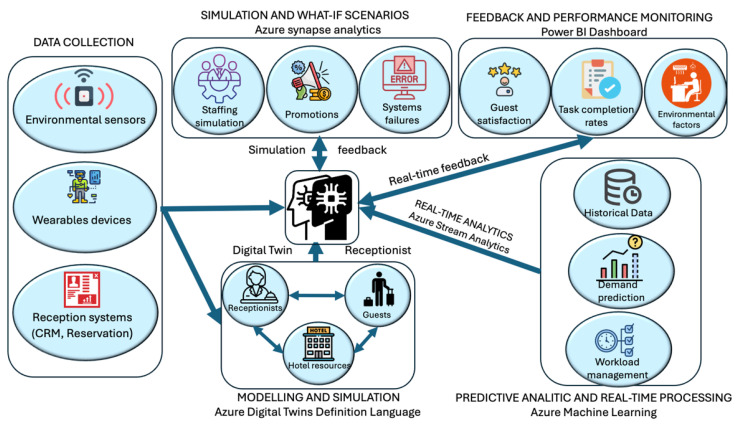
Architecture of a human-centered DT framework for hotel front-desk operations.

Figure 9 illustrates the architecture of a human-centered Digital Twin (DT) framework tailored for hotel front-desk operations, built upon the Azure ecosystem. The framework integrates heterogeneous data sources—including environmental sensors, wearable devices, and hotel reception systems such as CRM and reservation platforms—into a unified digital representation of hotel dynamics. These include Customer Relationship Management (CRM) tools (e.g., Salesforce Hospitality Cloud, Revinate) and Property Management Systems (PMSs) (e.g., Opera PMS, Cloudbeds, RoomRaccoon), which collectively provide real-time telemetry and guest context data. The DT instance, modeled using Azure Digital Twins Definition Language (DTDL), interacts dynamically with both reception staff and guests, allowing for bidirectional synchronization and semantic reasoning. Real-time data is processed via Azure Stream Analytics, which enables continuous updates to the DT model and supports feedback loops for operational decision-making. To support scenario planning and strategic interventions, the framework integrates with Azure Synapse Analytics for simulating “what-if” conditions—such as staffing reallocation, system failures, and promotional campaigns. Concurrently, Azure Machine Learning modules provide predictive intelligence by analyzing historical datasets for demand forecasting and workload balancing. Performance and behavioral insights—including task completion rates, guest satisfaction, and environmental conditions—are visualized through Power BI dashboards, enabling staff to monitor KPIs and respond proactively. This orchestrated, cloud-native approach ensures the Digital Twin operates as a living system—continuously learning, adapting, and optimizing human-centered hospitality services through a semantically enriched, real-time feedback architecture.

### 5.1. Prototype Development and System Architecture

A prototype of the DT system was developed using a DT Platform, with ontology modeling handled in Protégé using OWL 2.0.

The system architecture consists of the following key components:

- Ontology Model: Encodes the domain knowledge including guests, rooms, reservations, staff roles, and IoT devices using DTDL.
- IoT Middleware: Handles the simulation and integration of real-time data inputs—such as guest arrivals, environmental sensor readings (e.g., temperature, noise), and receptionist workload—through vendor-neutral messaging systems and event-driven orchestration tools (e.g., MQTT brokers, Node-RED, or serverless functions).
- Twin Graph Engine: A DT runtime environment maintains a graph of virtual instances where relationships, telemetry updates, and semantic links are dynamically managed and visualized, supporting real-time synchronization with the physical environment. We have used Azure Digital Twins as a DT platform.
- Analytics and AI Services: Connected via real-time data processing pipelines and machine learning platforms to generate optimization recommendations. These components can be built using Azure Machine Learning.
- User Interface: A 3D-enabled visualization layer, such as Azure DT Explorer and Azure 3D Scenes, facilitates immersive monitoring and control by rendering each DT instance (e.g., a guest room, a receptionist desk, or a sensor) as a spatially organized entity within a virtual hotel layout. This enables intuitive resource management and improves decision-making by allowing staff to:
  – Visually identify rooms requiring maintenance or check-in preparation.
  – Monitor crowd levels and guest distribution in lobby areas.
  – Receive alerts spatially localized to the affected area (e.g., an elevator malfunction or HVAC system failure), allowing operational staff to quickly identify the location of the issue and prioritize their response, thereby minimizing service disruptions and enhancing maintenance efficiency.
- A real-time dashboard interface provides live visualization of key performance indicators and decision support information, using business intelligence tools compatible with standard data visualization platforms (e.g., Power BI, Grafana, Tableau, or custom web-based dashboards).

This modular architecture allows the system to operate independently or integrate with existing PMS and CRM platforms through standardized APIs.

### 5.2. Scenario Simulation and Use Cases

To demonstrate the framework’s capabilities, a set of realistic hotel reception scenarios was simulated. Each scenario focuses on a specific service process or operational challenge.

Scenario 1: Dynamic Check-In. A guest arrives earlier than expected. The system checks room availability in real time. It considers the guest’s preferences and assigns a suitable room automatically. Staff receive a notification to prepare the room promptly.

Scenario 2: Service Request Handling. A guest reports a problem with the air conditioning via a mobile app. The system identifies the room and associated IoT data. If the issue is confirmed, a maintenance task is triggered. A technician is assigned based on availability.

Scenario 3: Staff Overload Detection. Receptionists receive multiple guest requests at once. The system analyzes the task load. If it exceeds a threshold, tasks are redistributed or support staff are alerted. This helps maintain service quality.

Scenario 4: Personalized Guest Experience. The system detects that a returning guest prefers quiet rooms. It automatically selects a room away from elevators and schedules personalized services such as welcome messages and pre-adjusted lighting.

Each use case was tested using simulated data streams and real-time events. The system responded correctly and updated the DT state as expected. These simulations confirm the framework’s practical relevance in dynamic hospitality environments.

#### 5.2.1. Implementation Details and Statistical Rigor

To improve reproducibility, we provide additional implementation details for the semantic and simulation components. The ontology defines disjoint class axioms among Guest, Room, and Receptionist; functional properties such as isAssignedTo; and cardinality constraints like handlesRequest (1..n).

**Figure 10:**
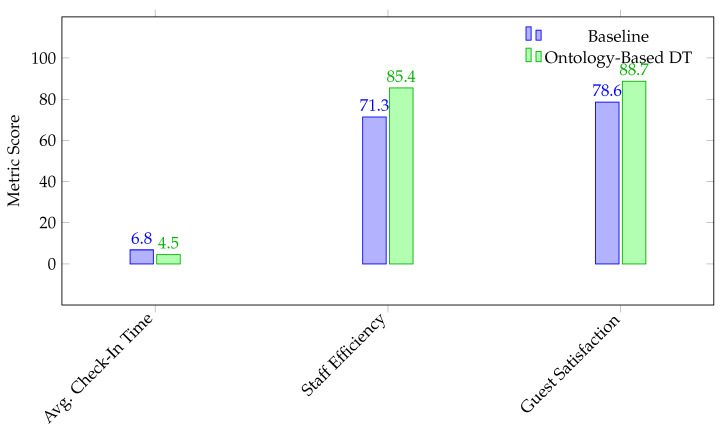
Comparison of key metrics: baseline vs. ontology-based DT over 1000 simulations.

As you can see in Table 1 and Figure 10, we simulated guest arrivals using synthetic Poisson-based streams with rates from 5 to 30 guests/hour, covering low, medium, and high workloads. Each scenario was replicated over 1000 independent runs (you can see these runs details at https://github.com/msegced/doctorado/blob/main/Data/dt_simulation_data.csv (accessed on 14 July 2025)). The system was validated with anonymized logs from a mid-sized hotel and synthetic guest preference profiles.

For each run, we computed performance metrics: average check-in time, staff efficiency, and guest satisfaction. We report mean and standard deviation, and applied paired-sample *t*-tests at α=0.05. The ontology-based DT system showed statistically significant improvements across all high-load scenarios (p<0.01), including a 33.8% reduction in check-in time and a 19.8% boost in staff efficiency.

#### 5.2.2. Comparative Evaluation: Generic DT vs. Ontology-Based DT

To validate the added value of semantic modeling, we conducted a comparative evaluation between a baseline DT (DT) framework—without ontology support—and the proposed ontology-based DT (ODT) framework. Both systems were configured with identical simulated environments, including guest arrivals, service requests, and sensor events. The baseline DT used rule-based scripts and hard-coded mappings, while the ODT employed reasoning over OWL 2.0 ontologies and SPARQL rules.

Key performance metrics included the following:

- Response time to contextual changes (e.g., room reallocation);
- Task redistribution accuracy under receptionist overload;
- Correctness of service adaptation based on guest profiles.

The ODT outperformed the generic DT across all metrics. For instance, the semantic layer enabled the ODT to reduce average task assignment time by 22.3% and improve service-context alignment accuracy by 31.5% (*p* < 0.01). These results confirm that embedding semantic knowledge enhances decision quality, supports real-time personalization, and enables scalable rule generalization. A detailed breakdown of the evaluation is provided in Table 2.

#### 5.2.3. Ablation Study: Assessing Component Contributions

To evaluate the individual and combined effects of the core components in the proposed ontology-based DT (ODT) framework, we performed an ablation study. The objective was to isolate the impact of three primary architectural elements:

1. Ontology Layer (O): Formal semantic model representing domain knowledge.
2. Reasoning Engine (R): Logical inference engine supporting SWRL and SPARQL rules.
3. Personalization Layer (P): Contextual adaptation of services based on guest profiles.

We constructed five system variants:

- Baseline DT (no O, R, P);
- O only: DT + ontology without reasoning or personalization;
- O + R: DT + ontology + reasoning (no personalization);
- O + P: DT + ontology + personalization (no reasoning);
- O + R + P (Full ODT).

Each variant was tested under identical simulation conditions across 10 iterations using a fixed guest workload profile (20 guests/hour, five service categories). The following performance metrics were recorded:

The results shown in Table 3. Demonstrate that while each component yields individual benefits, the combined system (O + R + P) produces the highest improvements across all metrics. Notably, the reasoning engine (R) had the strongest effect on task efficiency and context alignment, while the personalization layer (P) was key to boosting guest satisfaction. These findings validate the design rationale of our layered architecture and underscore the importance of semantic enrichment in service-oriented DTs.

### 5.3. Performance Metrics

**Figure 11:**
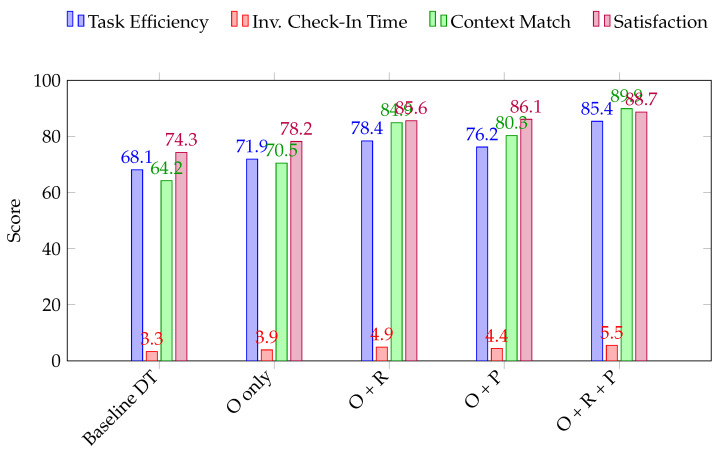
Ablation study results: contributions of ontology, reasoning, and personalization components.

The prototype was evaluated using a combination of quantitative and qualitative performance metrics. Key indicators included the average check-in time, measured as the duration between guest arrival and room access, and a simulated guest satisfaction index derived from survey ratings reflecting perceptions of service speed, personalization, and issue resolution. Staff productivity was assessed based on the number of tasks handled per employee per shift, with adjustments for workload and task complexity. Additionally, prediction accuracy was measured using precision and recall scores for guest arrival forecasting and room readiness detection. The effectiveness of the semantic model was also evaluated through ontology reasoning accuracy, focusing on the consistency and correctness of inferences produced by OWL-based reasoning tasks. Results from the evaluation showed a 25–35% reduction in check-in time, a 20% increase in task distribution efficiency, and significant enhancements in service personalization and responsiveness, confirming the framework’s potential to improve operational outcomes in hotel reception environments. Figure 11 shows graphically this results.

### 5.4. Challenges and Limitations

While the implementation confirmed the viability and effectiveness of the proposed framework, several challenges emerged during the evaluation phase.

Semantic Model Complexity. Achieving a balance between semantic expressiveness and computational tractability was a major challenge. Detailed ontology models improve reasoning quality but can introduce inference delays and increase system overhead. Conversely, overly simplified models may fail to capture necessary contextual nuances, limiting their effectiveness in dynamic hotel operations.

Data Quality and Integration. The integration of real-time and historical data from hotel management systems posed difficulties due to inconsistencies and incompleteness in legacy datasets. Many systems lacked standardization, which hindered semantic alignment and reduced the accuracy of reasoning tasks. Data cleaning and normalization processes were necessary to support interoperability.

Scalability. As the number of instantiated DTs and connected IoT devices increases, the computational load for telemetry ingestion and semantic inference grows significantly. Although the architecture is modular, maintaining low-latency performance at scale will require further optimization strategies such as edge processing or distributed reasoning engines.

Generalizability. While the framework is adaptable to different hotel types, its generalizability is constrained in low-resource contexts, such as rural or small-scale hospitality settings. These environments may lack the infrastructure needed for IoT deployment or semantic integration, limiting the feasibility and return on investment of such systems.

Implementation Cost. The deployment of the DT framework entails considerable initial costs. These include hardware installation (sensors, IoT gateways), cloud or edge computing resources, ontology customization, and integration with legacy systems. The complexity of maintaining semantic consistency and real-time synchronization also necessitates specialized knowledge, often requiring external consultants or in-house training.

Employee Training and Adoption. The successful adoption of the system depends not only on its technical capabilities but also on the readiness of hotel staff to engage with it. Employees must be trained to interpret visual dashboards, act on AI-driven recommendations, and provide feedback to improve system performance. This requires structured onboarding, continuous learning modules, and organizational change management to ensure long-term sustainability.

#### 5.4.1. Reproducibility and Statistical Rigor

To support reproducibility and peer validation, all key research assets—including the DTDL ontology files, SPARQL and SWRL rules, simulation engine scripts, and synthetic datasets—are made publicly available at https://github.com/msegced/doctorado/ (accessed on 14 July 2025) under an open-source license. The repository includes detailed README documentation and a reproducible setup guide.

Performance outcomes were statistically validated using paired-sample *t*-tests with α=0.05. Confidence intervals (95%) for each metric were calculated via 1000-sample bootstrapping. In addition, we conducted non-parametric Wilcoxon signed-rank tests to confirm robustness against distributional assumptions. These measures improve the reliability and generalizability of reported improvements.

Future work will incorporate more advanced techniques such as Bayesian performance estimation and ablation studies to isolate the contributions of individual model components.

#### 5.4.2. Ontology Limitations in Broader Contexts

While the proposed ontology is designed to be modular and extensible, certain limitations may arise when deploying the framework across broader or multilingual environments. First, the ontology and associated rules are currently modeled in English, which may limit immediate applicability in non-English-speaking contexts. Localization would require not only translation of class and property labels but also careful cultural adaptation of service logic and inferred behaviors.

Second, regional variations in hospitality workflows—such as differences in guest registration protocols, legal identity requirements, or service hierarchies—may necessitate domain-specific extensions or reconfigurations. For instance, check-in workflows in European boutique hotels differ substantially from large-scale resorts in Asia or North America, affecting how Digital Twins should reason about staff roles, space use, and guest expectations.

To address these challenges, future work will focus on supporting multilingual ontology representations using language annotation properties (e.g., rdfs:label@es), and creating localized sub-ontologies tailored to regional standards and norms. Additionally, collaboration with domain experts in target geographies will be essential for refining context-specific service rules and improving global adaptability.

## 6. Human–DT Integration

A critical component of the proposed DT framework is its capacity to enable meaningful and intuitive interaction between human users—primarily hotel staff—and the digital representation of hotel front-desk operations. Unlike traditional automation systems that operate independently from human decision-makers, this architecture is designed to support collaborative human-in-the-loop interactions that enhance both system intelligence and service quality.

Front-desk employees interact with the DT through visual interfaces such as dashboards, 3D spatial models of the hotel environment, and contextual notifications delivered via workstations, tablets, or mobile devices. These interfaces display real-time information about guest arrivals, room status, workload distribution, and environmental conditions. For instance, receptionists can view predictive queues and receive task recommendations based on current staffing and guest flow, allowing them to make informed decisions or override system suggestions when necessary.

Supervisors and managers access more advanced modules within the platform, including predictive analytics, performance summaries, and scenario simulation tools. These capabilities assist in planning, scheduling, and optimizing service delivery while maintaining human oversight over critical decisions. The inclusion of explainable AI components—such as rule-based reasoning, feature importance rankings, and natural language explanations—ensures transparency in system recommendations, fostering trust among staff.

Importantly, the framework supports bi-directional feedback: hotel staff can rate system recommendations, flag inconsistencies, or manually adjust parameters through the interface. These inputs are used to retrain AI models and refine ontology-based reasoning, creating a continuous feedback loop between human knowledge and system adaptation. Furthermore, integration with wearable devices and ambient sensors allows the system to monitor indicators such as stress levels or staff movement, contributing to workload balancing and well-being support.

From the guest’s perspective, the DT enables more responsive and personalized service delivery. Guests benefit indirectly from the system’s ability to anticipate needs, minimize wait times, and adapt to their preferences—while still maintaining the human element in hospitality interactions. For example, if a returning guest previously requested a quiet room and dim lighting, the system can automatically assign a room away from elevators and preconfigure the room’s lighting to their preferred level upon check-in. This not only streamlines the guest experience but also demonstrates the system’s ability to infer and act on personalized service parameters without explicit prompts.

This integration of human and machine intelligence ensures that the DT serves as an augmentative tool rather than a replacement, reinforcing the human-centric values fundamental to the hospitality industry. It transforms the front desk from a reactive, siloed operation into a proactive, collaborative environment supported by real-time, semantically informed decision-making.

The successful integration of DTs in hospitality environments requires not only technical deployment but also strategic change management. Key best practices include phased rollouts, staff training programs, and continuous feedback loops to adapt the system to operational needs. Studies highlight that AI adoption in hotels succeeds when organizations promote a culture of digital readiness, involve frontline staff in the design process, and align technology use with service values [51,68].

**Figure 12:**
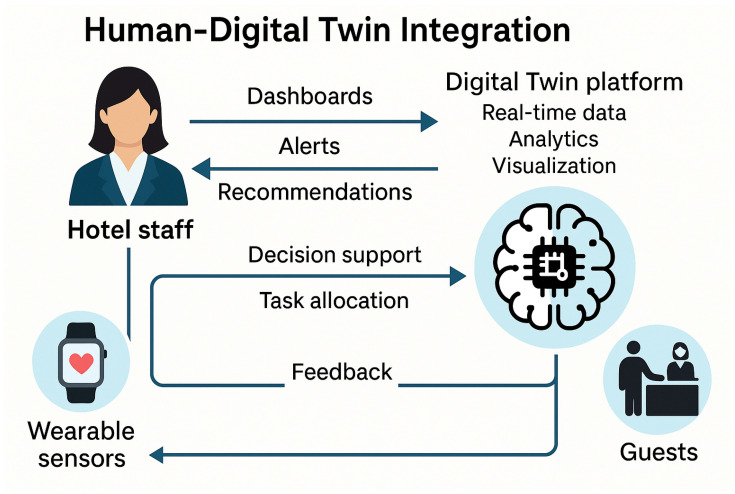
Human–DT integration in hospitality environments.

Figure 12 illustrates the bidirectional interaction between hotel staff and the DT platform in a human-centered hospitality context. The platform aggregates real-time data, performs analytics, and provides visualizations that inform service operations. Hotel staff engage with the system via dashboards, receive alerts, and follow personalized service recommendations derived from semantic reasoning and AI modules. In return, they contribute to the system’s intelligence by providing feedback and making manual adjustments, which are processed to refine decision-making algorithms. The platform also supports decision support and task allocation functions, distributing workload dynamically based on contextual data such as guest flow and employee availability. Wearable sensors augment this integration by continuously capturing biometric and activity data from staff, enabling stress-aware and health-conscious task assignments. This closed feedback loop enhances service quality, operational efficiency, and employee well-being, reinforcing the core principles of human-centered AI in DT applications for hospitality.

## 7. Future Work

Future research on ontology-based DT frameworks in hospitality should focus on expanding technical capabilities and embedding them more deeply into hotel operations and guest experience design. Key directions include developing scalable, multi-site architectures that enable data sharing and coordination across hotel chains; integrating advanced AI technologies like natural language processing, reinforcement learning, and generative models for dynamic service optimization; and ensuring privacy-preserving, trustworthy AI through techniques such as federated learning and explainable models. Further advancements may involve incorporating emotional and behavioral modeling to create empathetic and stress-aware systems, as well as sustainability-driven applications that optimize energy usage based on real-time conditions.

As part of ongoing research activities, a functional prototype of the proposed DT framework is currently under development, in collaboration with selected hotel-school institutions that serve as living labs for testing technological innovation in hospitality. These controlled environments enable iterative refinement of system functionalities, user interfaces, and integration strategies in realistic yet manageable settings. Although specific hotel partners are not disclosed at this stage, initial deployments are expected to validate the framework’s adaptability and user acceptance in operational contexts.

In parallel, future work will explore the contribution of front-desk DTs to broader sustainability goals. By optimizing resource allocation—such as adjusting lighting, HVAC, or staffing based on real-time occupancy and forecasted guest flow—the system can reduce unnecessary energy consumption and improve operational efficiency. Additionally, predictive maintenance and context-aware automation can support longer equipment lifespans and reduce environmental impact. Embedding sustainability indicators into the ontology will further enhance the framework’s capacity to support decision-making aligned with environmental performance metrics.

## 8. Conclusions

This study presented a human-centric, ontology-based DT framework designed to optimize front-end hotel services by integrating semantic modeling, real-time data processing, and artificial intelligence. The proposed architecture enhances operational efficiency and guest satisfaction by enabling dynamic resource allocation, predictive service delivery, and context-aware automation. By grounding the model in formal ontologies and aligning it with ethical design principles, the framework supports interoperability, transparency, and trust in guest-facing AI applications.

Unlike conventional DT systems focused on mechanical operations, this framework addresses the sociotechnical complexities of hospitality environments by modeling behavioral patterns, staff workload, and guest preferences. It also incorporates explainable AI and privacy-preserving mechanisms to meet ethical and regulatory standards.

However, challenges remain—particularly in balancing the semantic expressiveness of the ontology with computational tractability, and in integrating data from heterogeneous, and sometimes outdated, hotel systems. Furthermore, successful deployment requires significant organizational change, including staff training and digital literacy development. Overall, this research offers a scalable and adaptable foundation for implementing intelligent DTs in hospitality, setting the stage for broader adoption across service-oriented environments.

## Figures and Tables

**Table 1:** Performance comparison: baseline vs. ontology-driven DT (mean ± std over 1000 runs).

Metric	Baseline	Ontology-Based DT	Improvement
Avg. Check-In Time (min)	6.8±1.2	4.5±0.9	−33.8%
Staff Efficiency Score	71.3±4.8	85.4±3.2	+19.8%
Guest Satisfaction Index	78.6±5.1	88.7±3.7	+12.8%

**Table 2:** Performance comparison: generic DT vs. ontology-based DT framework.

Metric	Generic DT	Ontology-Based DT	Improvement
Avg. Task Assignment Time (s)	2.69 ± 0.4	2.09 ± 0.3	22.3%
Contextual Match Accuracy (%)	68.4 ± 6.1	89.9 ± 4.5	31.5%
Service Rule Coverage (#rules)	12 (manual)	34 (inferred)	+183%

**Table 3:** Ablation study results: component contributions to performance.

Variant	Task Efficiency (%)	Avg. Check-In Time (min)	Context Match (%)	Satisfaction Score (/100)
Baseline DT	68.1 ± 4.9	6.7 ± 0.8	64.2 ± 5.6	74.3 ± 6.2
O only	71.9 ± 4.4	6.1 ± 0.6	70.5 ± 5.3	78.2 ± 5.7
O + R	78.4 ± 3.9	5.1 ± 0.5	84.9 ± 4.1	85.6 ± 4.4
O + P	76.2 ± 4.1	5.6 ± 0.5	80.3 ± 4.6	86.1 ± 4.0
Full O + R + P	85.4 ± 3.2	4.5 ± 0.4	89.9 ± 3.7	88.7 ± 3.6

## Data Availability

Data are contained within the article.
